# Viscoelastic Parameters for Quantifying Liver Fibrosis: Three-Dimensional Multifrequency MR Elastography Study on Thin Liver Rat Slices

**DOI:** 10.1371/journal.pone.0094679

**Published:** 2014-04-10

**Authors:** Maxime Ronot, Simon A. Lambert, Mathilde Wagner, Philippe Garteiser, Sabrina Doblas, Miguel Albuquerque, Valérie Paradis, Valérie Vilgrain, Ralph Sinkus, Bernard E. Van Beers

**Affiliations:** 1 Department of Radiology, Beaujon University Hospitals Paris Nord Val de Seine, Clichy, France; 2 University Paris Diderot, Sorbonne Paris Cité, France; 3 IPMA, INSERM U773, Centre de Recherche Biomédicale Bichat-Beaujon, Paris, France; 4 Department of Pathology, Beaujon University Hospitals Paris Nord Val de Seine, Clichy, France; Sezione di Gastroenterologia, Italy

## Abstract

**Objective:**

To assess in a high-resolution model of thin liver rat slices which viscoelastic parameter at three-dimensional multifrequency MR elastography has the best diagnostic performance for quantifying liver fibrosis.

**Materials and Methods:**

The study was approved by the ethics committee for animal care of our institution. Eight normal rats and 42 rats with carbon tetrachloride induced liver fibrosis were used in the study. The rats were sacrificed, their livers were resected and three-dimensional MR elastography of 5±2 mm liver slices was performed at 7T with mechanical frequencies of 500, 600 and 700 Hz. The complex shear, storage and loss moduli, and the coefficient of the frequency power law were calculated. At histopathology, fibrosis and inflammation were assessed with METAVIR score, fibrosis was further quantified with morphometry. The diagnostic value of the viscoelastic parameters for assessing fibrosis severity was evaluated with simple and multiple linear regressions, receiver operating characteristic analysis and Obuchowski measures.

**Results:**

At simple regression, the shear, storage and loss moduli were associated with the severity of fibrosis. At multiple regression, the storage modulus at 600 Hz was the only parameter associated with fibrosis severity (r = 0.86, p<0.0001). This parameter had an Obuchowski measure of 0.89+/−0.03. This measure was significantly larger than that of the loss modulus (0.78+/−0.04, p = 0.028), but not than that of the complex shear modulus (0.88+/−0.03, p = 0.84).

**Conclusion:**

Our high resolution, three-dimensional multifrequency MR elastography study of thin liver slices shows that the storage modulus is the viscoelastic parameter that has the best association with the severity of liver fibrosis. However, its diagnostic performance does not differ significantly from that of the complex shear modulus.

## Introduction

Over the past decade, various studies have shown the usefulness of ultrasound and MR elastography for the assessment of the severity of liver fibrosis [1]–[12]. Most of these studies have been focused on the determination of liver stiffness, based on the measurement of the speed of the mechanical waves propagating through the tissue. However, several other viscoelastic parameters may be calculated. With three-dimensional MR elastography the complete wave field can be measured in the liver, enabling the calculation of the complex shear modulus, G*, reflecting tissue stiffness, but also the storage modulus, Gd, reflecting the elasticity, and the loss modulus, Gl, related to the viscosity [13]. Moreover, with multifrequency MR elastography, the exponent of the frequency power law (γ), can be calculated and reflects wave scattering related to the architectural organization of the tissue [14]. The usefulness of these different viscoelastic parameters for assessing the severity of fibrosis has not been much explored [10], [15], [16].

Most of the elastography studies were performed in humans using histological scores on liver biopsies as reference methods [17]. The main limitations of these studies are the acquisition of MR images with low spatial resolution, the presence of respiratory artifacts, the sampling variability of liver biopsies and the use of semi-quantitative histological scores [18]. Few teams have reported studies performed in-vivo on animal models [9], [16], and the same limitations remain. To address these concerns, we performed in this study high-resolution three-dimensional multifrequency MR elastography examinations on thin rat liver slices. Thin liver slices are increasingly used as ex-vivo models to study liver diseases [19], [20]. Moreover, to obtain “pure” liver fibrosis, without associated cholestastis or steatois, we induced fibrosis by chronic administration of carbon tetrachloride [21].

The purpose of our study was thus to assess in a high-resolution model of thin liver rat slices which viscoelastic parameter at three-dimensional multifrequency MR elastography has the best diagnostic performance for quantifying liver fibrosis.

## Materials and Methods

### Ethics statement

This study was carried out in strict accordance with the European guidelines of Care and Use of Laboratory Animals. The protocol was approved by the local Committee on the Ethics of Animal Experiments (“Bichat-Debré” Animal Ethics Committee; Number: 2011-15/773-0065). Euthanasia was performed after anesthesia of the rats with a mixture of 4% isoflurane in oxygen (Arkema, Paris, France) to minimize animal suffering.

### Experimental model of liver fibrosis

The study group included 50 male Wistar rats aged 8 weeks and weighting 252±28 g. The rats were housed by groups of three in stainless steel wire cages and maintained at mean temperature of 21±1°C. The cages were placed in a room with a 12-hour light-dark cycle. The rats had free access to standard food and water. Eight rats were used as controls and liver fibrosis was induced in the 42 other rats. No rat died before imaging.

Fibrosis was induced by twice-weekly intraperitoneal injections of 0.1 ml/100 g carbon tetrachloride diluted at 50% in olive oil vehicle. The rat population was divided in seven groups of six rats. The first group of rats was injected with carbon tetrachloride during two weeks, the second during three weeks, and so on until the last cohort was injected during eight weeks, to analyze fibrosis progression on a weekly basis. Each week, a group of six rats was sacrificed. The sacrifice was obtained three days after the last injection of carbon tetrachloride to decrease liver inflammation. Euthanisia was performed with intraperitoneal injection of 1 ml of sodium thiopental (Abbott Laboratories, Chicago, IL) after anesthesia of the rats with a mixture of 4% isoflurane in oxygen (Arkema, Paris, France).

**Figure 1:**
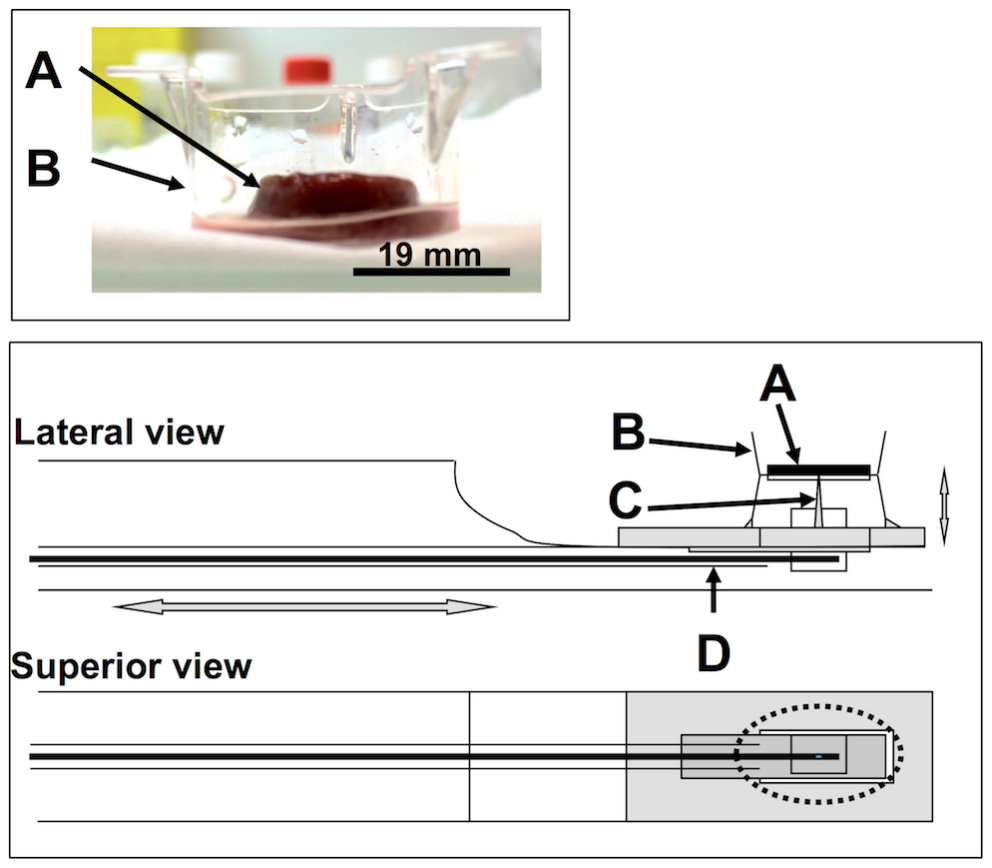
Details of the homemade support used for the MR elastography examinations. The picture on the top shows a thin liver sample (A) placed in a plastic insert (B). The bottom illustration shows the way the sample is placed on the homemade support. The vibration is transmitted by a toothpick placed in the center of the liver sample (C). An electromagnetic shaker located outside the MR scanner (not shown) is used to generate mechanical vibrations via a flexible carbon fiber rod to the toothpick (D).

The liver was removed and a cylindrical hepatic sample was obtained with a 19 mm diameter punch (C.S. Osborne & Co, Harrison, NJ). A thin (5±2 mm) slice was then cut out of this cylindrical sample, and placed in a 22 mm cell culture insert (Millicell, EMD Millipore Corporation, Billerica, MA) containing physiological medium to maintain a constant level of moisture within the liver sample (Fig. 1). Temperature was monitored during the whole MR elastography experiment to eliminate its potential influence on the measured viscoelastic parameters.

### MR elastography

MR elastography was performed in a horizontal-bore 7-T MR imaging system (Pharmascan, Bruker, Erlangen, Germany) with 80 mm diameter volume coil for emission and a decoupled 18 mm diameter surface coil for reception. An electromagnetic shaker (Brüel & Kjaer, Nærum, Denmark) located outside the MR scanner was used to transmit mechanical vibrations via a flexible carbon fiber rod to a toothpick placed in the center of the liver sample and attached to the bench (Fig. 1). The insert was also attached to the bench, but independent from the toothpick holder.

T2-weighted transverse images of each sample were obtained for proper slice positioning of the MR elastography acquisition. A steady-state MR elastography sequence was used in the three spatial directions of motion to obtain volumetric images of the three-dimensional propagating wave inside the sample. The MR elastography acquisitions were obtained sequentially with three different mechanical excitation frequencies of 500, 600, and 700 Hz. The in-plane spatial resolution was 390 μm and the slice thickness 400 μm. The MR image acquisition characteristics are detailed in Table 1.

**Table 1:** MR acquisition parameters.

	T2-weighted		MR elastography	
**Field of view (mm)**	25×25		25×25	
**Matrix (phase × frequency encoding)**	256×256		64×64	
**Mechanical excitation frequency (Hz)**	-	500	600	700
**Repetition time (ms)**	2500	604	503	571
**Echo time (ms)**	11	30	26	23
**Number of signals acquired**	-	8	8	8
**Number of spatial dimensions acquired**	-	3	3	3
**Section thickness (μm)**	400		400	
**Intersection gap (mm)**	0		0	
**In-plane spatial resolution (μm)**	98		390	
**Number of sections**	7		7	
**Acquisition time (s)**	32		2160^a^	

The total acquisition time corresponds to the sum of the three MR elastography experiments.

**Figure 2:**
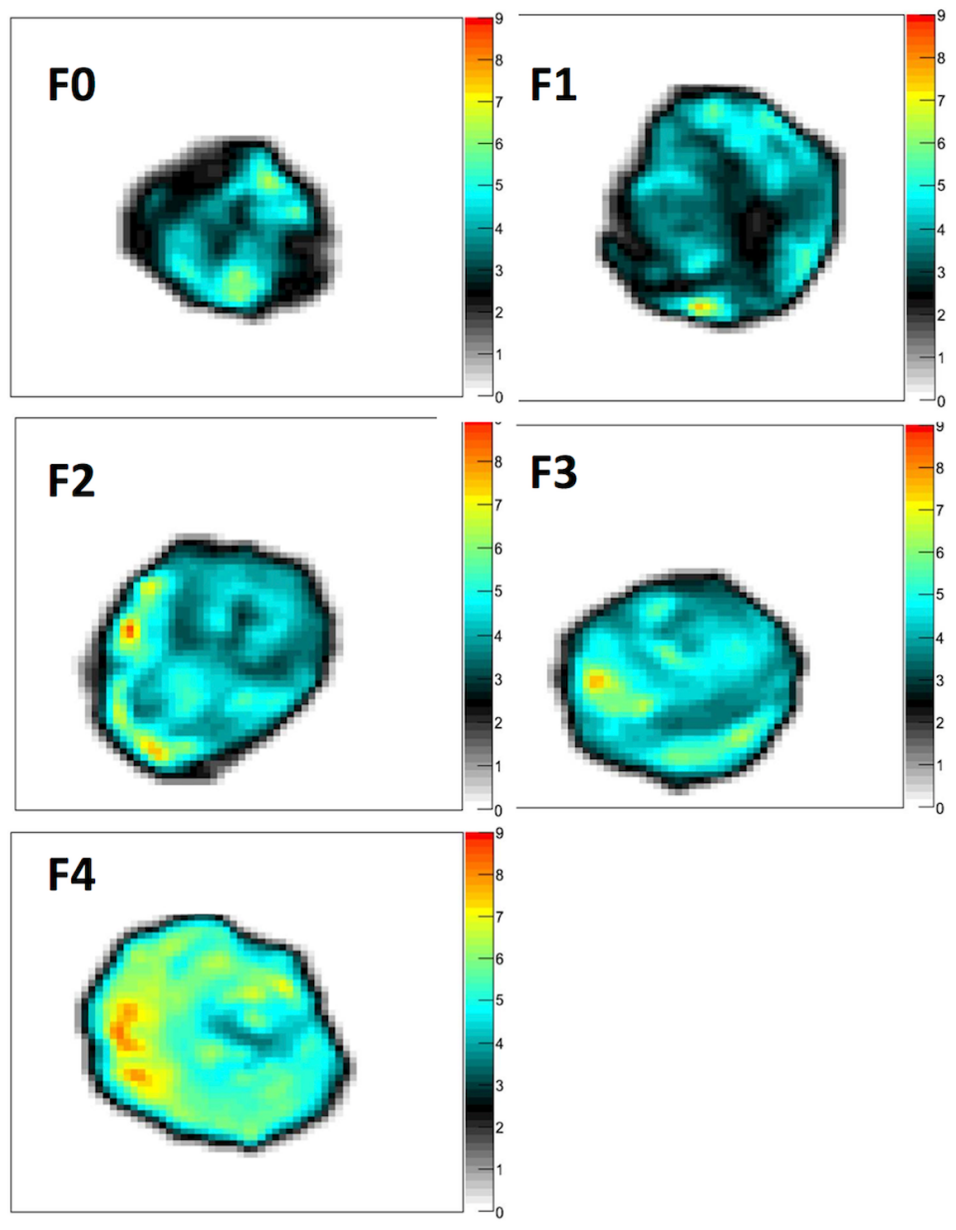
Parametric maps of Gd at 600 Hz performed on thin liver slices with fibrosis ranging from F0 to F4. With increasing fibrosis, Gd increases as shown by the progressive changes in colors of the parametric maps.

The moduli G*, Gd and Gl (kPa) were calculated on a pixel-per-pixel basis and displayed on parametric maps by fitting a polynomial function to the displacement values and inverting the local time-harmonic wave equation, as previously described [14]. Regions of interest encompassing the whole sample sections, but avoiding their borders and the vibration point, were drawn on the parametric maps to obtain mean values of G*, Gd, and Gl (Fig. 2). Moreover, the relation between the viscoelastic parameters (G*, Gd, Gl) and the excitation frequency was fitted on a power law and the exponent of the law (γ) was extracted [14]. The post-processing of the MR elastography images was performed by a radiologist with 7-year experience in abdominal radiology. The radiologist was unaware of the results of histology. Before starting the study, the reproducibility of the method was evaluated by performing three different viscoelastic parameter measurements in a paraffin phantom. Similar analysis was performed in the rats with normal liver.

### Histological analysis

After the MR elastography examination, all samples were fixed in formalin, embedded in paraffin and stained with hematoxylin-eosin, picrosirius red and Masson trichrome. The histological slides were analyzed by a pathologist with 15-year experience in liver pathology and who was blinded to the imaging data. Fibrosis, necrosis and inflammation were assessed with the METAVIR score [22]. Fibrosis stage (F) was scored as F0 (absent), F1 (portal fibrosis), F2 (portal fibrosis with few septa), F3 (septal fibrosis), and F4 (cirrhosis). Necro-inflammatory activity (A) was graded as A0 (absent), A1 (mild), A2 (moderate), or A3 (severe). Steatosis was graded according to Brunt et al. [23].

Quantitative morphometry was performed to determine the area of fibrosis (sample area stained with picrosirius red relative to the total sample area) with an image segmentation software after converting the glass slides into digital slides (ImageScope, Aperio, Vista, CA) [18].

### Statistical analysis

Values are expressed as means and standard deviations or percentages, as appropriate. The reproducibility of the visco-elastic measurements was assessed with coefficients of variation.

Differences in MR elastography viscoelastic parameters between the METAVIR stages were assessed with analysis of variance (ANOVA). The relation between histological and the viscoelastic parameters were performed with simple and multiple regressions. The discriminative ability of the viscoelastic parameters for the identification of the stages of fibrosis was assessed by receiver operating characteristic (ROC) curve analysis and expressed as areas under the ROC curve (AUROCs).

Given the fact that the METAVIR score is a non-binary ordinal-scale gold standard, we also calculated the Obuchowski measures [24]. These measures are a multinomial version of AUROCs. With Obuchowski measures, a weighting scheme is used, based on a reference distribution of fibrosis stages. In our study, the reference distribution of fibrosis stages was chosen according to Payan et al. as F0: 6%, F1: 39%, F2: 28%, F3: 14%, F4: 13% [25]. With the Obuchowski measure, each pairwise comparison is also weighted by a penalty function proportional to the difference in METAVIR units between stages [24]. The Obuchowski measure represents the probability that the viscoelastic parameters will correctly rank two randomly chosen liver samples from different fibrosis stages according to the weighting scheme, with a penalty for misclassifying the samples. Comparison between the Obuchowski measures of the different viscoelastic parameters was made with DeLong test.

A p-value≤0.05 was considered significant for all tests. The analyses were performed with the Statistical Package for the Social Sciences (SPSS) software (version 20.0, SPSS Inc., Chicago, IL).

## Results

### Pathological and morphometric analyses

Out of the 50 rats, three (6%) were excluded from the analysis because of technical problems during the MR examinations. A total of 47 rats was analyzed. Histological data are detailed in Table 2. Briefly, 8 rats (17%) had F0 fibrosis stage, 8 (17%) F1, 8 (17%) F2, 8 (17%) F3 and 15 (32%) F4. Necro-inflammatory activity was graded as A0 in 8 rats (17%), A1 in 31 (66%), and A2 in 8 (17%).

**Table 2:** METAVIR score and fibrosis area of the rat population (N = 47).

	F0 (N = 8)	F1 (N = 8)	F2 (N = 8)	F3 (N = 8)	F4 (N = 15)
**A0 (N = 8)**	8	0	0	0	0
**A1 (N = 31)**	0	4	7	8	12
**A2 (N = 8)**	0	4	1	0	3
**Area of fibrosis (mean% ± SD)**	0.33±0.16	0.64±0.17	1.27±0.06	2.1±0.7	4.85±2.27

The mean area of fibrosis assessed with morphometry was 2.29±2.26% for the whole cohort. Details according to the fibrosis stage are given in Table 2. The correlation between the fibrosis stages and the fibrosis areas was excellent (r = 0.96, p<10^-4^). No rat developed steatosis.

### Relation between MR elastography parameters and fibrosis extent

In the phantoms, all viscoelastic parameters were extracted with coefficients of variation<1%. In rats with normal liver, the viscoelastic parameters were extracted with coefficients of variation<2%.

**Figure 3:**
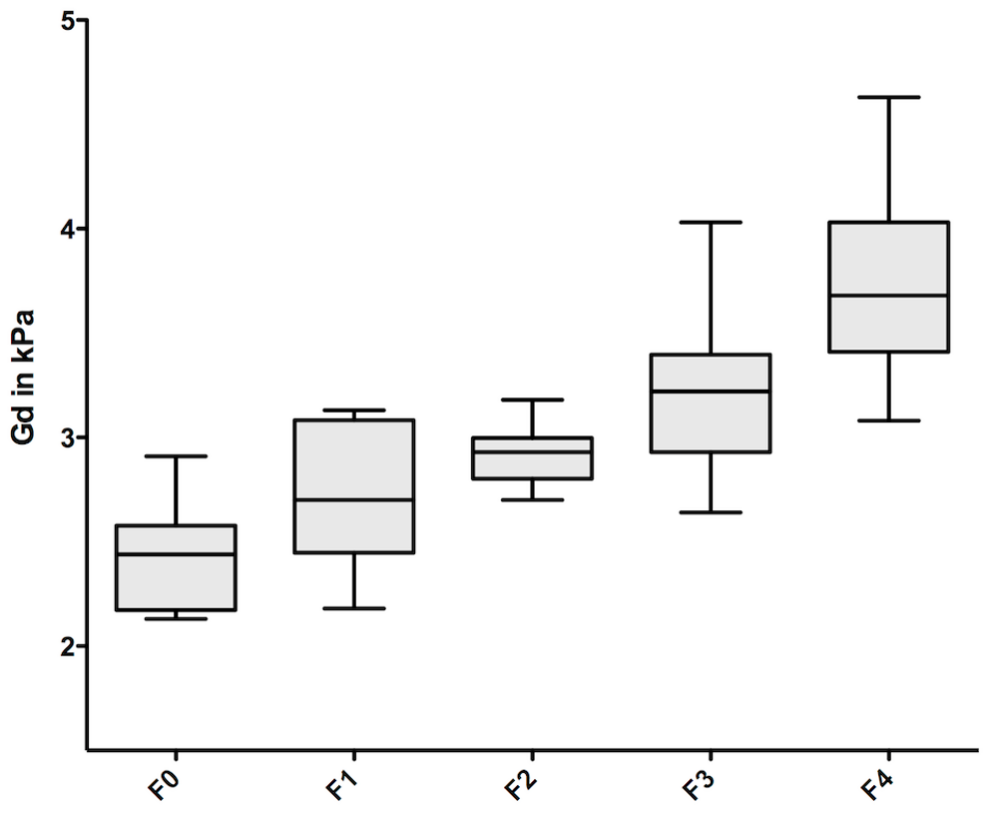
Distribution of Gd (kPa) according to METAVIR stages of fibrosis with mechanical excitation of 600 Hz. There is a progressive increase of Gd according to the METAVIR stage. Line within boxes represents median, lower and upper limits of boxes represent 25 and 75 percentiles respectively. Whiskers represent 10 and 90 percentiles.

The viscoelastic parameters for the three mechanical frequencies are detailed in Table 3. The moduli G* and Gd increased significantly with the progression of fibrosis (ANOVA, p<10^-4^) (Fig. 3) at 500, 600 and 700 Hz. At 600 and 700 Hz, Gl showed no significant increase from F0 to F3, and a significant increase for F4 (p<10^−4^ and p = 0.002 for 600 Hz and 700 Hz, respectively). There was no significant increase of Gl at 500 Hz with increasing fibrosis.

**Table 3:** Details of the viscoelastic parameters according to the METAVIR score.

		Total population (N = 47)	F0 (N = 8)	F1 (N = 8)	F2 (N = 8)	F3 (N = 8)	F4 (N = 15)	ANOVA (p value)
	**500 Hz**	3.43±0.69	2.46±0.54	3.0±0.41	3.27±0.3	3.36±0.15	3.98±0.65	**<10^−4^**
G* (kPa)	**600 Hz**	3.66±0.67	2.92±0.26	3.2±0.36	3.43±0.15	3.73±0.39	4.4±0.36	**<10^−4^**
	**700 Hz**	4.73±0.79	3.96±0.42	4.2±0.49	4.45±0.23	4.88±0.38	5.56±0.64	**<10^−4^**
	γ	0.99±0.38	1.33±0.69	0.90±0.14	0.90±0.24	0.74±0.11	0.99±0.25	**0.031**
	**500 Hz**	2.95±0.6	2.48±0.40	2.6+/−0.40	2.8±0.30	3.3±0.40	3.4±0.60	**<10^−4^**
**Gd (kPa)**	**600 Hz**	3.15±0.6	2.43±0.26	2.73±0.35	2.92±0.15	3.2±0.42	3.8±0.44	**<10^−4^**
	**700 Hz**	4.1±0.70	3.3±0.40	3.7±0.40	3.8±0.20	4.2±0.40	4.9±0.60	**<10^−4^**
	γ	0.99±0.38	1.29±0.66	0.88±0.19	0.90±0.26	0.74±0.14	1.04±0.24	0.063
	**500 Hz**	1.5±0.3	1.4±0.40	1.5±0.20	1.4±0.20	1.5±0.30	1.7±0.30	0.066
**Gl (kPa)**	**600 Hz**	1.8±0.3	1.6±0.20	1.6±0.20	1.6±0.20	1.8±0.20	2.1±0.20	**<10^−4^**
	**700 Hz**	2.2±0.4	2.1±0.50	1.9±0.20	2.1±0.10	2.3±0.10	2.5±0.30	**0.002**
	γ	1.1±0.53	1.35±0.9	0.72±0.38	1.07±0.37	1.26±0.55	1.14±0.30	0.150

**Figure 4:**
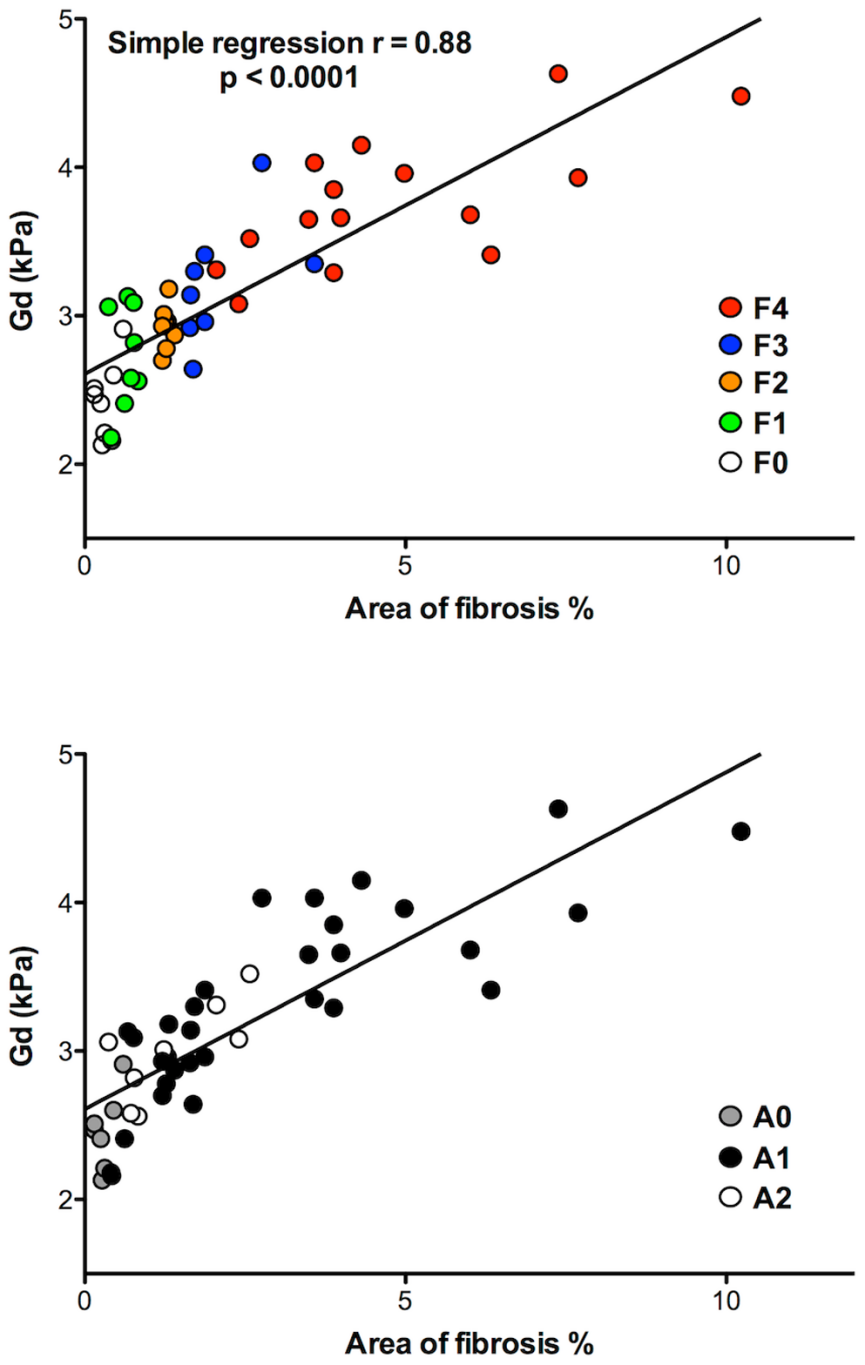
Correlation between Gd and the area of fibrosis with details of the METAVIR stages (A) and the necro-inflammatory activity (B).

At simple regression, there were significant relations between the METAVIR stage and all the viscoelastic parameters, except γ. The multiple linear regression identified Gd at 600 Hz as the only parameter related to the METAVIR score (r = 0.86, p<10^−4^) (Table 4). Similarly, there were significant correlations between the areas of fibrosis assessed with morphometry and all the viscoelastic parameters but γ (r = 0.56–0.86, p<10^−4^) (Table 4 and Fig. 4).

**Table 4:** Correlations between the viscoelastic parameters on one hand and the METAVIR scores and the percentages of fibrosis on the other.

		Correlation with METAVIR				Correlation with % of fibrosis			
		Simple regression		Multiple regression		Simple regression		Multiple regression	
		r	p	r	p	r	p	r	p
	**500 Hz**	0.75	**<10^−4^**			0.72	**<10^−4^**		
G* (kPa)	**600 Hz**	0.84	**<10^−4^**			0.84	**<10^−4^**		
	**700 Hz**	0.79	**<10^−4^**			0.81	**<10^−4^**		
	γ	−0.25	0.08			−0.14	0.17		
	**500 Hz**	0.77	**<10^−4^**			0.73	**<10^−4^**		
**Gd (kPa)**	**600 Hz**	0.86	**<10^−4^**	0.86	**<10^−4^**	0.86	**<10^−4^**	0.86	**<10^−4^**
	**700 Hz**	0.80	**<10^−4^**			0.80	**<10^−4^**		
	γ	−0.16	0.14			**−**0.08	0.38		
	**500 Hz**	0.40	0.004			0.56	**<10^−4^**		
**Gl (kPa)**	**600 Hz**	0.69	**<10^−4^**			0.77	**<10^−4^**		
	**700 Hz**	0.50	**<10^−4^**			0.60	**<10^−4^**		
	γ	0.03	0.42			−0.03	0.43		

There was no influence of the METAVIR necro-inflammatory activity on G*, (p = 0.21, p = 0.15, and p = 0.13 at 500, 600, and 700 Hz, respectively), Gd (p = 0.15, p = 0.10, and p = 0.11 at 500, 600, and 700 Hz, respectively) or Gl (p = 0.59, p = 0.43, and p = 0.23 at 500, 600, and 700 Hz, respectively).

### ROC curve analysis at 600 Hz

**Figure 5:**
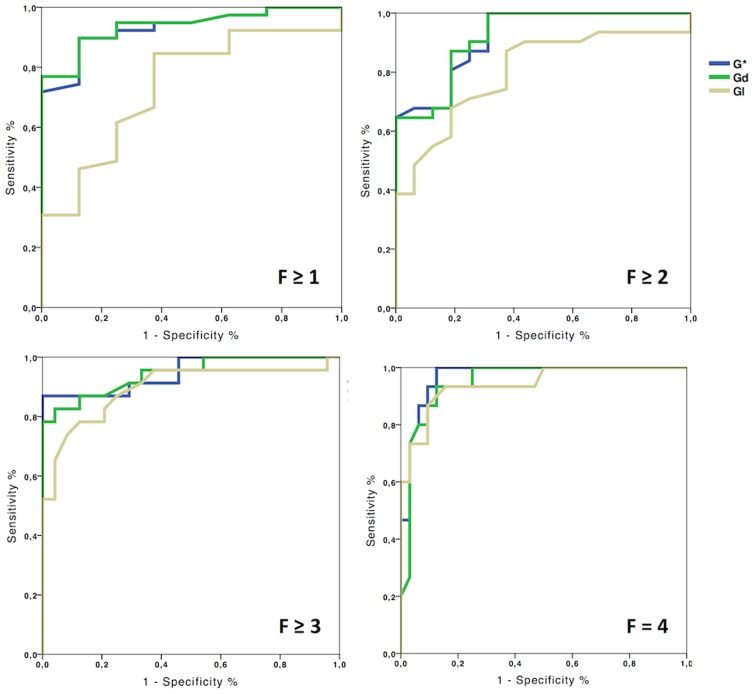
ROC curves of the moduli G*, Gd and Gl at 600 Hz for the differentiation of the METAVIR stages. The AUROCs of Gd are larger than 0.9.

The ROC curve analysis has been focused on the diagnostic performance at 600 Hz, as Gd at 600 Hz was found to be correlated with the METAVIR score and the area of fibrosis at multiple regression. The AUROCs of Gd ranged from 0.92±0.04 for F≥2 to 0.95±0.03 for F4 (Fig. 5). The Obuchowski measure for Gd was 0.89±0.03. It was significantly higher than that of Gl (0.78±0.04, p = 0.028) but not than that of G* (0.88±0.03, p = 0.84).

For the identification of fibrosis≥2, the cut-off value of 2.92 kPa for Gd at 600 Hz had sensitivity, specificity, positive and negative predictive values of 87%, 81%, 91%, and 73%, respectively.

For the identification of fibrosis = 4, the cut-off value of 3 .31 kPa for Gd at 600 Hz had sensitivity, specificity, positive and negative predictive values of 87%, 90%, 76%, and 93%, respectively.

## Discussion

The results of our study show that, in an ex-vivo model of thin liver slices, the viscoelastic parameters assessed with three-dimensional multifrequency MR elastography are substantially associated with the extent of fibrosis. Among these parameters, Gd, a parameter related to tissue elasticity, was the only factor identified at multiple regression. This parameter showed excellent performance in distinguishing between the different stages of fibrosis, as illustrated by its high Obuchowski measure. However, the diagnostic performance of Gd did not differ significantly from that of G*.

MR elastography has been shown to be useful for staging liver fibrosis in humans [8]–[12]. However, the studies are limited by several factors including the acquisition of MR images with low spatial resolution, the presence of respiratory artifacts, the sampling variability with small biopsies and the use of semi-quantitative histological scores [24]. Better correlations between imaging and pathology can be obtained when MR elastography is performed in small animal models of liver fibrosis. Indeed, in these models, semi-quantitative and quantitative measurements of fibrosis area in the whole liver can be obtained [12]. However, the problem of respiratory artifacts during MR elastography and the difficulty of precise matching the MR imaging and histological slices remain in animal studies.

In our study, we address these issues by showing the feasibility of performing three-dimensional multifrequency MR elastography in an ex-vivo model of thin slices of liver fibrosis. With this model, artifact-free, viscoelastic maps of the liver can be obtained with a high spatial resolution of 390×390×400 microns as shown in Fig. 2. This model is free from steatosis and cholestasis, thus offering an MR elastographic analysis of merely “pure” fibrosis, with some inflammation. Furthermore, the three-dimensional MR elastography used in our study is ideally suited to assess various viscoelastic parameters, including G* reflecting the stiffness, Gd reflecting the elasticity, and Gl related to the viscosity, by using an inversion algorithm of the complex wave equation [14). With this method, we observed that Gd was the best viscoelastic parameter to quantify liver fibrosis as it showed a substantial relation with both the METAVIR score and the area of fibrosis at morphometry.

If we observed a clear association between the viscoelastic parameters and the liver fibrosis stage, we did not observe such association between the viscoelastic parameters and the liver inflammation grade. In contrast to this last finding, liver inflammation has been reported to increase liver stiffness in previous studies performed with ultrasound elastography [26]. Our negative results regarding the influence of inflammation may be explained by the fact that most livers in our study had no or mild inflammation (83%). Only 17% of the livers had moderate inflammation and no case of severe inflammation was observed. This high proportion of livers with mild inflammation was intentionally obtained, as the rats were sacrificed three days after the end of the carbon tetrachloride intoxication to decrease inflammation and obtain a model of relatively “pure” fibrosis. Again, the aim of our study was not to assess the influence of inflammation on the viscoelastic parameters, but to assess which viscoelastic parameter had the best diagnostic performance to assess liver fibrosis.

In contrast to Gd, Gl did not increase until the fibrosis level F2, followed by an increase for major fibrosis (F3 and F4). Similar results, i.e. a slight increase of Gl until F3 and a higher increase for severe F4 fibrosis, have been recently published by Leclerc et al. in human fibrosis [27]. These results are in accordance with previously reported results, showing that Gd (related to the elasticity) was superior to Gl (related to the viscosity) to stage human liver fibrosis [15], [27]. In contrast to the observations in liver fibrosis, recent studies show that Gl is superior to Gd for determining the malignancy of liver tumors [13]. These findings show that different viscoelastic parameters that can be measured with three-dimensional MR elastography are of value for the assessment of different liver diseases.

However, in our study, the superiority of Gd relative to the other viscoelastic parameters for the correlations with the severity of liver fibrosis did not translate in an increased accuracy of Gd relative to G* for fibrosis staging. Indeed, the Obuchowski measure of Gd (0.89±0.03) was not significantly higher than that of G* (0.88±0.03). The shear modulus G* reflects tissue stiffness or its elasticity if one considers that the tissue is purely elastic (meaning that the viscosity equals zero). Tissue stiffness is also the only parameter than can be assessed with ultrasound or MR elastography methods based on the simple measurement of shear wave speed. Our results show that G* is an accurate parameter for determining the severity of liver fibrosis as shown by its high Obuchowski measure. This also implies that elastography methods that measure tissue stiffness by looking at wave speed are valid for assessing fibrosis severity.

We used a multifrequency approach that allows calculating the exponent of the frequency power law, γ, which reflects tissue geometry [14]. In our study, γ was not associated with the progression of fibrosis, and showed no significant variation between the different METAVIR groups. Similar results have been reported by Asbach et al. [10] in liver fibrosis in humans.

Two concerns may be raised in studies about fibrosis severity assessment, one regarding the use of semiquantitive histological scoring as reference method and the other regarding the use of conventional ROC analysis for determining accuracy [18], [24]. It has been suggested that the quantification of the area of fibrosis at morphometry is a better indicator of fibrosis severity than fibrosis staging with semiquantititave scoring systems. Indeed, these scoring systems such as the METAVIR score are considered to assess the architectural changes rather than measure the amount of fibrosis and are prone to intraobserver and interobserver variability [18]. However, in our study, we did not observe a difference in the strength of the relations between the viscoelastic parameters and those two references, namely morphometry and METAVIR scoring. This suggests that METAVIR scoring, when performed on sufficiently large tissue samples, remains an adequate method for assessing the severity of liver fibrosis [28].

For determining the accuracy of non-invasive fibrosis staging methods, the conventional ROC approach is used in most studies [8], [10]. This approach has been challenged for two reasons. First, the determination of the AUROC is based on the assumption that the reference examination is binary, whereas fibrosis staging uses an ordinal scale. Second, the proportion of each stage of fibrosis in the sample might not fit the distribution in the reference population to which the indices are applied. As a result, the comparison of different AUROCs based on samples with different stage distributions may be flawed. Obuchowski described a measure that overcomes these two methodological issues [29]–[31]. Therefore, we used the Obuchowski measure in our study. This measure confirmed the superiority of Gd over Gl, as the Obuchowski measure of Gl was significantly lower than that of Gd (0.78±0.04 versus 0.89±0.03, p = 0.028).

Our study has some limitations. First the carbon tetrachloride intoxication model leads to relatively small amounts of liver fibrosis, even after several weeks of injections. These amounts differ from those observed in human fibrosis and might lead to a bias in the MR elastography evaluation. Second, the viscoelastic properties of our ex vivo model may differ from those in vivo, mainly because of the absence of tissue perfusion ex-vivo. Nevertheless, the ex-vivo model might also help characterizing the effect of liver fibrosis severity on the viscoelastic parameters, without confounding effects.

In conclusion, our ex-vivo thin-liver slice rat model allowed precise analysis of liver viscoelastic parameters with three-dimensional multifrequency MR elastography at 7T. The storage modulus Gd was the viscoelastic parameter that had the best association with liver fibrosis severity but its diagnostic performance did not differ significantly from that of the complex shear modulus G*.

## References

[1] Castera L, Vergniol J, Foucher J, Le Bail B, Chanteloup E, et al (2005) Prospective comparison of transient elastography, Fibrotest, APRI, and liver biopsy for the assessment of fibrosis in chronic hepatitis C. Gastroenterology 128: 343–350. 15685546 10.1053/j.gastro.2004.11.018

[2] Ziol M, Handra-Luca A, Kettaneh A, Christidis C, Mal F, et al (2005) Noninvasive assessment of liver fibrosis by measurement of stiffness in patients with chronic hepatitis C. Hepatology 41: 48–54. 15690481 10.1002/hep.20506

[3] Castera L (2012) Noninvasive methods to assess liver disease in patients with hepatitis B or C. Gastroenterology 142: :1293–1302 e4. 10.1053/j.gastro.2012.02.017 22537436

[4] Marcellin P, Ziol M, Bedossa P, Douvin C, Poupon R, et al (2009) Non-invasive assessment of liver fibrosis by stiffness measurement in patients with chronic hepatitis B. Liver Int 29: 242–247. 18637064 10.1111/j.1478-3231.2008.01802.x

[5] Oliveri F, Coco B, Ciccorossi P, Colombatto P, Romagnoli V, et al (200 8) Liver stiffness in the hepatitis B virus carrier: a non-invasive marker of liver disease influenced by the pattern of transaminases. World J Gastroenterol 14: 6154–6162. 18985805 10.3748/wjg.14.6154 PMC2761576

[6] Friedrich-Rust M, Nierhoff J, Lupsor M, Sporea I, Fierbinteanu-Braticevici C, et al (2012) Performance of Acoustic Radiation Force Impulse imaging for the staging of liver fibrosis: a pooled meta-analysis. J Viral Hepat 19: e212–219. 22239521 10.1111/j.1365-2893.2011.01537.x

[7] Ferraioli G, Tinelli C, Dal Bello B, Zicchetti M, Filice G, et al (2012) Accuracy of real-time shear wave elastography for assessing liver fibrosis in chronic hepatitis C: a pilot study. Hepatology 56: 2125–2133. 22767302 10.1002/hep.25936

[8] Huwart L, Sempoux C, Vicaut E, Salameh N, Annet L, et al (2008) Magnetic resonance elastography for the noninvasive staging of liver fibrosis. Gastroenterology 135: 32–40. 18471441 10.1053/j.gastro.2008.03.076

[9] Yin M, Woollard J, Wang X, Torres VE, Harris PC, et al (2007) Quantitative assessment of hepatic fibrosis in an animal model with magnetic resonance elastography. Magn Reson Med 58: 346–353. 17654577 10.1002/mrm.21286

[10] Asbach P, Klatt D, Schlosser B, Biermer M, Muche M, et al (2010) Viscoelasticity-based staging of hepatic fibrosis with multifrequency MR elastography. Radiology 257: 80–86. 20679447 10.1148/radiol.10092489

[11] Venkatesh SK, Yin M, Ehman RL (2013) Magnetic resonance elastography of liver: Technique, analysis, and clinical applications. J Magn Reson Imaging 37: 544–555. 23423795 10.1002/jmri.23731 PMC3579218

[12] Salameh N, Peeters F, Sinkus R, Abarca-Quinones J, Annet L, et al (2007) Hepatic viscoelastic parameters measured with MR elastography: correlations with quantitative analysis of liver fibrosis in the rat. Journal of magnetic resonance imaging. J Magn Reson Imaging 26: 956–962. 17896384 10.1002/jmri.21099

[13] Garteiser P, Doblas S, Daire JL, Wagner M, Leitao H, et al (2012) MR elastography of liver tumours: value of viscoelastic properties for tumour characterisation. Eur Radiol 22: 2169–2177. 22572989 10.1007/s00330-012-2474-6

[14] Sinkus R, Siegmann K, Xydeas T, Tanter M, Claussen C, et al (2007) MR elastography of breast lesions: understanding the solid/liquid duality can improve the specificity of contrast-enhanced MR mammography. Magn Reson Med 58: 1135–1144. 17969009 10.1002/mrm.21404

[15] Huwart L, Sempoux C, Salameh N, Jamartt J, Annet L, et al (2007) Liver fibrosis: noninvasive assessment with MR elastography versus aspartate aminotransferase-to-platelet ratio index. Radiology 245: 458–466. 17940304 10.1148/radiol.2452061673

[16] Salameh N, Larrat B, Abarca-Quinones J, Pallu S, Dorvillius M, et al (2009) Early detection of steatohepatitis in fatty rat liver by using MR elastography. Radiology 253: 90–97. 19587308 10.1148/radiol.2523081817

[17] Ishak K, Baptista A, Bianchi L, Callea F, De Groote J, et al (1995) Histological grading and staging of chronic hepatitis. J Hepatol 22: 696–969. 7560864 10.1016/0168-8278(95)80226-6

[18] Standish RA, Cholongitas E, Dhillon A, Burroughs AK, Dhillon AP (2006) An appraisal of the histopathological assessment of liver fibrosis. Gut 55: 569–578. 16531536 10.1136/gut.2005.084475 PMC1856155

[19] Starkel P, Leclercq IA (2001) Animal models for the study of hepatic fibrosis. Best Pract Res Clin Gastroenterol 25: 319–333. 10.1016/j.bpg.2011.02.004 21497748

[20] Lagaye S, Shen H, Saunier B, Nascimbeni M, Gaston J, et al (2012) Efficient replication of primary or culture hepatitis C virus isolates in human liver slices: a relevant ex vivo model of liver infection. Hepatology 56: 861–872. 22454196 10.1002/hep.25738

[21] Hadi M, Westra IM, Starokozhko V, Dragovic S, Merema MT, et al (2013) Human precision-cut liver slices as an ex vivo model to study idiosyncratic drug-induced liver injury. Chem Res Toxicol 26: 710–720. 23565644 10.1021/tx300519p

[22] Bedossa P, Poynard T (1996) An algorithm for the grading of activity in chronic hepatitis C. The METAVIR Cooperative Study Group. Hepatology 24: 289–293. 8690394 10.1002/hep.510240201

[23] Brunt EM, Janney CG, Di Bisceglie AM, Neuschwander-Tetri BA, Bacon BR, et al (1999) Nonalcoholic steatohepatitis: a proposal for grading and staging the histological lesions. Am J Gastroenterol 94: 2467–2474. 10484010 10.1111/j.1572-0241.1999.01377.x

[24] Lambert J, Halfon P, Penaranda G, Bedossa P, Cacoub P, et al (2008) How to measure the diagnostic accuracy of noninvasive liver fibrosis indices: the area under the ROC curve revisited. Clin Chem 54: 1372–1378. 18539647 10.1373/clinchem.2007.097923

[25] Payan C, Roudot-Thoraval F, Marcellin P, Bied N, Duverlie G, et al (2005) Changing of hepatitis C virus genotype patterns in France at the beginning of the third millenium: The GEMHEP GenoCII Study. J Viral Hepat 12: 405–413. 15985012 10.1111/j.1365-2893.2005.00605.x

[26] Yoshioka K (2013) How to adjust the inflammation-induced overestimation of liver fibrosis using transient elastography? Hepatol Res 43: 182–184. 23409851 10.1111/j.1872-034X.2012.01096.x

[27] Leclerc GE, Charleux F, Robert L, Ho Ba Tho MC, Rhein C, et al (2013) Analysis of liver viscosity behavior as a function of multifrequency magnetic resonance elastography (MMRE) postprocessing. J Magn Reson Imaging 38: 422–428. 23293060 10.1002/jmri.23986

[28] Bedossa P, Dargere D, Paradis V (2003) Sampling variability of liver fibrosis in chronic hepatitis C. Hepatology 38: 1449–1457. 14647056 10.1016/j.hep.2003.09.022

[29] Obuchowski NA (2005) Estimating and comparing diagnostic tests' accuracy when the gold standard is not binary. Acad Radiol 12: 1198–1204. 16099683 10.1016/j.acra.2005.05.013

[30] Obuchowski NA (2006) An ROC-type measure of diagnostic accuracy when the gold standard is continuous-scale. Stat Med 25: 481–493. 16287217 10.1002/sim.2228

[31] Obuchowski NA, Goske MJ, Applegate KE (2001) Assessing physicians' accuracy in diagnosing paediatric patients with acute abdominal pain: measuring accuracy for multiple diseases. Stat Med 20: 3261–3278. 11746317 10.1002/sim.944

